# Toxicity Assessment of Refill Liquids for Electronic Cigarettes

**DOI:** 10.3390/ijerph120504796

**Published:** 2015-04-30

**Authors:** Vincent Varlet, Konstantinos Farsalinos, Marc Augsburger, Aurélien Thomas, Jean-François Etter

**Affiliations:** 1 Unit of Toxicology, University Center of Legal Medicine, 1000 Lausanne 25, Switzerland; E-Mails: vincent.varlet@chuv.ch (V.V.); marc.augsburger@chuv.ch (M.A.); aurelien.thomas@chuv.ch (A.T.); 2 Department of Cardiology, Onassis Cardiac Surgery Center, Kallithea 17674, Greece; E-Mail: kfarsalinos@gmail.com; 3 Department of Pharmacology, University of Patras, Patras 26500, Greece; 4 Swiss Center of Applied Human Toxicology, University of Lausanne, 1000 Lausanne 25, Switzerland; 5 Institute of Global Health, Faculty of Medicine, University of Geneva, 1211 Geneva, Switzerland

**Keywords:** electronic cigarette, electronic nicotine delivery systems, e‑cigarette, e‑liquid, quality control.

## Abstract

We analyzed 42 models from 14 brands of refill liquids for e-cigarettes for the presence of micro-organisms, diethylene glycol, ethylene glycol, hydrocarbons, ethanol, aldehydes, tobacco-specific nitrosamines, and solvents. All the liquids under scrutiny complied with norms for the absence of yeast, mold, aerobic microbes, *Staphylococcus aureus*, and *Pseudomonas aeruginosa*. Diethylene glycol, ethylene glycol and ethanol were detected, but remained within limits authorized for food and pharmaceutical products. Terpenic compounds and aldehydes were found in the products, in particular formaldehyde and acrolein. No sample contained nitrosamines at levels above the limit of detection (1 μg/g). Residual solvents such as 1,3-butadiene, cyclohexane and acetone, to name a few, were found in some products. None of the products under scrutiny were totally exempt of potentially toxic compounds. However, for products other than nicotine, the oral acute toxicity of the e-liquids tested seems to be of minor concern. However, a minority of liquids, especially those with flavorings, showed particularly high ranges of chemicals, causing concerns about their potential toxicity in case of chronic oral exposure.

## 1. Introduction

Electronic cigarettes (e-cigarettes) are increasingly popular [1,2]. They comprise a battery-powered atomizer that produces vapor for inhalation from cartridges or tanks that usually contain propylene glycol or glycerol (or a mix of both), flavors, nicotine, water and ethanol [3]. Surveys show that 11% to 21% of adult smokers in the United States report having ever used e-cigarettes, which translates into several millions users [4,5,6,7]. Laboratory testing has shown that some refill liquids (e‑liquids) for e-cigarettes contain impurities and toxic substances, or are not filled true to label [2], although a recent report showed that the quality of most e-liquids is compliant with norms [8]. Another concern is the lack of mandatory manufacturing standards for e-cigarettes and e-liquids. There are many manufacturers, but few, if any products are manufactured along standards imposed on medications. There is no guarantee that e‑liquids do not contain impurities or toxic components. In addition, recent reports have revealed an increasing number of cases of accidental exposure to e-liquids, mainly through ingestion, and a few fatal cases were reported in the press, although not as case reports in peer-reviewed literature [9]. Nicotine may also oxidize in open containers to produce degradation products within the liquid itself, leading to the unintentional presence of products due to degradation processes in liquid refills [10].

Although vapors of e-cigarettes seem to be less toxic than tobacco smoke, relatively little is known about the content and toxicity of these vapors and of the liquids used to produce these vapors [2]. Thus, the objectives of this study were to assess levels of chemical and biological constituents, including micro-organisms, diethylene glycol, ethylene glycol, hydrocarbons, ethanol, aldehydes, tobacco-specific nitrosamines, and solvents, in a large set of commercial e‑liquids purchased on the Internet (Table 1).

## 2. Material and Methods

Previous research enabled us to identify the most popular brands of e-liquids used in several countries (USA, UK, France, Switzerland) [8,11,12]. We selected the brands that dominate the market in the USA and much of Western Europe, and we selected several other brands for convenience (e.g., from websites that sent products to Switzerland). We analyzed 42 bottles of 14 different brands purchased on the Internet in 2013 and received by mail. Upon receipt in Geneva, the bottles were kept at room temperature and protected from the light until they were sent for analysis to Helvic Laboratories (Stoke-on-Trent, UK) for the microbiological tests and to Hall Analytical Laboratories (Manchester, UK) for the chemical analyses. Analyses of ethylene glycol, conducted at a later time point by Hall Analytical Laboratories, were conducted on 32 bottles of the same 14 brands (Table 2). The liquids were kept at room temperature by these laboratories from the reception of the products to the analyses, which were performed in 2013–2014.

**Table 1:** Characteristics of 42 bottles of liquids for e-cigarettes, 2013.

Brand	Model	Code	PG or VG	Bottle Capacity (mL)	Nicotine Label (mg/mL)		Batch Number “Exp” or “Use by” Date	Ordered on Website	Country Sent from
Janty	Classic Sahara, High “GSH H”	H60339	PG	10	16		Exp 10 December 2014	Eu.jantyworld.com	France
Janty	Vitaya eLiquid Texas, high	H60340	PG	10	16		Pro: 22 November 2012 Exp: 21 November 2013	Eu.jantyworld.com	France
Janty	Elixir PG eLiquid Havana, high	H60341	PG	15	16		Pro: January 2013 Exp: January 2015	Eu.jantyworld.com	France
Janty	Elixir VG eLiquid Tobacco	H60342	VG	15	16		Pro: November 2012 Exp: November 2014.	Eu.jantyworld.com	France
Janty	Elixir VG Spearmint	H60343	VG	15	16		Pro: November 2012 Exp: November 2014.	Eu.jantyworld.com	France
Janty	Elixir VG Texas	H60344	VG	15	16		Pro: November 2012 Exp: November 2014	Eu.jantyworld.com	France
Janty	Elixir VG Golden Sahara	H60345	VG	15	16		Pro: May 2012. Exp: May 2014	Eu.jantyworld.com	France
Ecigexpress	Minty Menthol	H60346	PG	10	18		N/A	Ecigexpress.com	USA
Ecigexpress	Full Flavor Tobacco VG edit	H60347	VG	10	18		N/A	Ecigexpress.com	USA
Ecigexpress	e-Juice Red USA Mix	H60348	PG	10	24		N/A	Ecigexpress.com	USA
Vapor4Life	Gunslinger Nobacco Juice USA	H60349	PG + VG	30	18		V0021001840. Best used by 31 December 2012	Vapor4life.com	USA
Vapor4Life	VG 555 WOW Vapor Juice	H60350	VG	30	36		PRD: 30 December 2012. Exp: 30. December 2014.	Vapor4life.com	USA
Vapor4Life	Wowboy Peppermint Wow Vapor Juice	H60351	N/A	30	18		PRD: 30. December 2012. Exp: 30. December 2014.	Vapor4life.com	USA
Totally Wicked	American red tobacco	H60352	PG + VG	10	6		Lot # TW1215	totallywicked-eliquid.co.uk	UK
Totally Wicked	Titanium Ice	H60353	VG	50	72		Batch No. NGLY034	totallywicked-eliquid.co.uk	UK
Totally Wicked	Platinum Ice	H60354	VG	50	54		Batch No. NGLY0034	totallywicked-eliquid.co.uk	UK
Totally Wicked	Glycerine (to dilute «Ice» models)		VG					totallywicked-eliquid.co.uk	UK
Sedansa	American Blend Premium	H60355	PEG 400	10	0		N/A	Sedansa.be	Belgium
Sedansa	Mint Premium	H60356	VG	10	0		N/A	Sedansa.be	Belgium
Sedansa	Turkish Tobacco Premium	H60357	PG + VG	10	0		N/A	Sedansa.be	Belgium
Sedansa	7 Star	H60358	N/A	10	0		N/A	Sedansa.be	Belgium
Sedansa	RY4	H60359	N/A	10	0		N/A	Sedansa.be	Belgium
Johnson Creek	Domestic	H60360	PG + VG	15	18		Lot B1221811	johnsoncreeksmokejuice.com	USA
Johnson Creek	JC Original	H60361	PG + VG	15	18		Lot B1131801	johnsoncreeksmokejuice.com	USA
Johnson Creek	Espresso	H60362	PG + VG	15	18		Lot B0221807	johnsoncreeksmokejuice.com	USA
Johnson Creek	Read Oak Tennessee Cured	H60363	VG	15	18		Lot B1231817	johnsoncreeksmokejuice.com	USA
TECC Titan fluid	American red high	H60364	PG	10	18		April 2013–October 2014	Theelectroniccigarette.co.uk	UK
TECC Titan	Apple, High Blended	H60365	N/A	10	18		March 2013–September 2014	Theelectroniccigarette.co.uk	UK
TECC Titan	Virginia high	H60366	N/A	10	18		April 2013–October 2014	Theelectroniccigarette.co.uk	UK
TECC Titan	Cappucino high	H60367	N/A	10	18		March 2012–September 2013	Theelectroniccigarette.co.uk	UK
TECC Titan	American red Super High	H60368	N/A	10	36		April 2013–October 2014	Theelectroniccigarette.co.uk	UK
Intellicig	ECOpure E-Liquid	H60369	VG	10		Rich 45	Lot: ZBAX Use by April 2015	Intellicig.com	UK
e-cigarettes.fr	Kyozen. E-liquide MLB high	H60370	PG + VG	10		18	N/A	e-cigarettes-fr	France
e-cigarettes.fr	Kyozen. E-liquide Sahara high	H60371	PG + VG	10		18	N/A	e-cigarettes-fr	France
e-cigarettes.fr	Kyozen. E-liquide Ruyan n° 4 high	H60372	PG + VG	10		18	N/A	e-cigarettes-fr	France
CigLib	Tobacco, fort 16 mg/mL	H60373	N/A	10		16	February 2012–August 2013	Ciglib.fr	France
V2 Cigs	V2 Platinum	H60374	PG + VG	25		18	Batch YSBA Best before 30 May 2015	V2cigs.com	USA
e-liquide.com	Liqua Mints	H60375	PG + VG	10		18	Batch B133 Exp: December 2014.	e-liquide.com	France
Tasty Vapor	Amaretto Stone Sour	H60376	30% VG	29.6 mL		18	Born on 6 June 2013	Tastyvapor.us	USA
Tasty Vapor	Black Licorice	H60377	30% VG	29.6 mL		18	Born on 6 June 2013	Tastyvapor.us	USA
Tasty Vapor	Apple Pie Candy	H60378	30% VG	29.6 mL		18	Born on 6 June 2013	Tastyvapor.us	USA
e-cig.com	“LIQ” e-liquid base VG	H60379	VG	50		48	N/a	e-cig.com	China
e-cig.com	Pure nicotine “LIQ”	H60380	VG	10		18	N/a	e-cig.com	China

PG: propylene glycol; VG: vegetable glycerine; PEG: Polythylene glycol; PRO/PRD: date produced; EXP: date expires; N/A: not available.

**Table 2:** Content of 42 bottles of liquids for e-cigarettes, 2013.

Brand	Model	Code	Diethylene Glycol (μg/g)	Ethylene Glycol (μg/g)	Hydrocarbons, (μg/g)	Ethanol (μg/g)
Janty	Classic Sahara, High “GSH H”	H60339	<0.5	9.27	<25	--
Janty	Vitaya eLiquid Texas, high	H60340	<0.5	6.19	<25	399
Janty	Elixir PG eLiquid Havana, high	H60341	<0.5	4.29	<25	60
Janty	Elixir VG eLiquid Tobacco	H60342	<0.5	6.59	<25	241
Janty	Elixir VG Spearmint	H60343	<0.5	7.02	790 (Limonene)	118
Janty	Elixir VG Texas	H60344	<0.5	7.57	<25	445
Janty	Elixir VG Golden Sahara	H60345	1.6	6.70	<25	1157
Ecigexpress	Minty Menthol	H60346	<0.5	--	1829 (Limonene)	2455
Ecigexpress	Full Flavor Tobacco VG edition	H60347	0.6	2.91	<25	--
Ecigexpress	e-Juice Red USA Mix	H60348	<0.5	--	<25	1931
Vapor4Life	Gunslinger Nobacco Juice USA	H60349	<0.5	--	<25	1196
Vapor4Life	VG 555 WOW Vapor Juice	H60350	<0.5	--	<25	-
Vapor4Life	Wowboy Peppermint Wow Juice	H60351	<0.5	4.12	<25	-
Totally Wicked	American red tobacco	H60352	<0.5	6.49	<25	548
Totally Wicked	Titanium Ice, nicotine base	H60353	<0.5	3.60	<25	-
Totally Wicked	Platinum Ice, nicotine base	H60354	<0.5	2.90	<25	-
Sedansa	American Blend Premium	H60355	<0.5	4.80	<25	390
Sedansa	Mint Premium	H60356	<0.5	5.72	<25	31
Sedansa	Turkish Tobacco Premium	H60357	<0.5	3.68	<25	375
Sedansa	7 Star	H60358	<0.5	--	<25	-
Sedansa	RY4	H60359	<0.5	--	<25	-
Johnson Creek	Domestic	H60360	4.0	9.43	<25	1211
Johnson Creek	JC Original	H60361	0.6	20.28	<25	359
Johnson Creek	Espresso	H60362	<0.5	--	<25	212
Johnson Creek	Read Oak Tennessee Cured	H60363	3.5	17.71	<25	1840
TECC Titan fluid	American red high	H60364	<0.5	4.17	<25	-
TECC Titan fluid	Apple, High Blended	H60365	<0.5	4.02	<25	336
TECC Titan fluid	Virginia high	H60366	0.8	--	<25	2915
TECC Titan fluid	Cappucino high	H60367	<0.5	4.75	<25	6
TECC Titan fluid	American red Super High	H60368	<0.5	--	<25	-
Intellicig	ECOpure E-Liquid	H60369	1.0	7.48	<25	3453
e-cigarettes.fr	E-liquide MLB high	H60370	<0.5	5.79	<25	1695
e-cigarettes.fr	E-liquide Sahara high	H60371	<0.5	3.78	<25	2074
e-cigarettes.fr	E-liquide Ruyan n° 4 high	H60372	<0.5v	--	<25	2694
CigLib	Tobacco, fort 16 mg/mL	H60373	<0.5	6.03	<25	-
V2 Cigs	V2 Platinum	H60374	<0.5	5.13	<25	1335
e-liquide.com	Liqua Mints	H60375	<0.5	4.48	779 (Limonene)	-
Tasty Vapor	Amaretto Stone Sour	H60376 *	<0.5	4.40	106,479	404
						
Tasty Vapor	Black Licorice	H60377 **	<0.5	4.20	2082	3675
Tasty Vapor	Apple Pie, Candy	H60378	<0.5	--	<25	77
e-cig.com	“LIQ” e-liquid base VG	H60379	2.2	66.97	<25	2250
e-cig.com	Pure nicotine “LIQ”	H60380	0.8	6.12	<25	3623

* Sample H60376: alpha-Pinene (isomer) (4790 µg/g); beta-pinene (isomer) (27,137 µg/g); limonene (50,936 µg/g); 1,4-Cyclohexadiene, 1-methyl-4-(1-methylethyl) [gamma terpinene] (11,438 µg/g); benzene, 1-methyl-2-(1-methylethyl)-[para-cymene] (5498 µg/g); cyclohexane, 1-methyl-4-(5-methyl-1-methylene-4-hexenyl)-(6950 µg/g). ** Sample H60377: alpha-pinene (641 µg/g) and limonene (1441 µg/g).

### 2.1. Microbiological Tests

We tested the e-liquids for the absence of *Staphylococcus aureus* and *Pseudomonas aeruginosa* according to methodology described in the European Pharmacopoeia Section 2.6.13, and proceeded to microbial enumeration for total aerobic microbial count (TAMC) and total yeast and mold count (TYMC) according to the methodology described in the European Pharmacopeia Section 2.6.12 [13]. These tests are required by the European Pharmacopoeia for oromucosal products*.* For inhalation use and for aqueous preparations intended for oral use, TAMC should be ≤100 colony forming unit (CFU) per mL and TYMC should be ≤10 CFU/mL [13]. The liquids were diluted at 1:100 for the TAMC and TYMC tests, but we report results for the undiluted concentrations. For microbiological tests only, two batches of each liquid (purchased at different dates and with different batch numbers) were analyzed.

### 2.2. Chemical Tests

For each e-liquid tested, diethylene glycol and hydrocarbons analyses were performed after methanolic dilution via gas chromatography-mass spectrometry (GC-MS), and ethylene glycol analyses were performed via chemical ionisation GC-MS (selected ion monitoring). Solvents and ethanol analyses were done through headspace GC-MS, and tobacco-specific nitrosamines analyses (TSNA) through liquid chromatography coupled to tandem mass spectrometry (LC-MS/MS). For aldehydes monitoring, a known sample weight of each sample was placed directly onto a LpDNPH tube and eluted with 5 mL of acetonitrile, then analysed by LC coupled with ultra-violet detection and MS (LC-UV/MS). The reference solutions, used for identification and quantification of the substances, contained known levels of each substance under scrutiny.

### 2.3. Toxicity Assessment

We determined whether the concentrations of each of the molecules detected in the liquids were within a normal range for food or pharmaceutical products, based on the ICH guidelines for new drug products, the European Pharmacopoeia for active ingredients, and other relevant literature [13,14].

We also assessed the conformity of the e-liquids by comparing the observed concentrations to the acceptable limits defined in the strictest food residue regulations available [15], and to the standards for good manufacturing practices (GMP) used in the flavor and fragrance industry [16].

To assess the potential toxicity of the e-liquids, we compared the concentrations measured to parameters available for human exposure in the environment (air, water) or in food: Estimated Human Exposure (EHE), Acceptable Daily Intake (ADI), Maximized Survey-Derived Intake (MSDI), and Tolerable Daily Intake (TDI). For the conformity assessment of toxicity, we selected the lowest values of acceptance available (in the EU, US or in various national regulations such as Germany, Japan and France) to investigate the toxicity of e-liquids. Two separate assessments were performed: (a) potential acute oral toxicity was assessed following a hypothetical scenario of ingestion of 10 grams of liquid; (b) potential chronic toxicity associated with an assumed average daily consumption of 3 grams of e-liquid. The daily consumption of 3 grams was based on evidence from surveys of dedicated e-cigarette users [17].

## 3. Results

### 3.1. Microbiological Analyses

All the liquids under scrutiny complied with European Pharmacopoeia norms for the absence of *Staphylococcus aureus* and *Pseudomonas aeruginosa*. Four samples had total aerobic microbial count = 1 CFU/mL (H60348, H60355, H60357 and H60379). All the other investigated samples had total aerobic microbial count <1 CFU/mL. All the samples except one had total yeast and mold counts <1 CFU/mL. A glycerin bottle for mixing the liquids, purchased from *Totally Wicked*, had total yeast and mold count = 1 CFU/mL.

### 3.2. Diethylene Glycol

All the samples analyzed had concentrations of diethylene glycol below 4 µg/g (the limit of detection (LOD) was 0.5 µg/g).

### 3.3. Ethylene Glycol

With the exception of 3 samples (H60361: 20.3 µg/g, H60363: 17.7 µg/g, and H60379: 67 µg/g), all samples contained less than 10 μg/g ethylene glycol.

### 3.4. Hydrocarbons

The concentrations of hydrocarbons were below the LOD of 25 µg/g for all except five samples (H60344: 790 µg/g, H60346: 1830 µg/g, H60375: 780 µg/g, H60376: > 100,000 µg/g and H60377: 2080 µg/g). Most of these hydrocarbons were terpenic compounds, which were probably used as flavoring agents. Limonene was identified in all these cases as the main component, followed by pinene isomers and gamma-terpinene (Table 2).

### 3.5. Ethanol

All the samples had concentrations of ethanol below 3.7 mg/g.

### 3.6. Aldehydes

Formaldehyde concentrations ranged from 0.1 to 9.0 µg/g and acetaldehyde concentrations from 0.05 to 10.2 µg/g (Table 3). Acrolein content was below the limit of detection (LOD: 0.111 µg/g) in all except three products (H60360: 0.18 µg/g, and H60363: 0.21 µg/g and H60380: 1.03 µg/g). Propionaldehyde was below the LOD (0.043 µg/g) in all except seventeen products (Table 3). Butyraldehyde was below the LOD (0.077 µg/g) in all except eight products. Valeraldehyde (LOD: 0.281 µg/g) and 2,5-dimethylbenzaldehyde (LOD: 0.027 µg/g) were below their respective LOD in all products. Crotonaldehyde was below the LOD (0.053 µg/g) in all except two products (H60363: 0.067 µg/g, and H60377: 0.084 µg/g). For benzaldehyde, twelve products were below the LOD (0.035 µg/g) (Table 3). Isovaleraldehyde levels were below the LOD (0.194 µg/g) in all except four samples: H60349 (1.54 µg/g), H60351 (1.09 µg/g), H60360 (3.14 µg/g), and H60379 (1.43 µg/g). *O*-tolualdehyde levels were under the LOD (0.017 µg/g) except in one product (H60378: 0.043 µg/g), and m- and p-tolualdehyde levels, measured as the sum of these two isomers, were below the LOD (0.018 µg/g) except for H60377 (0.069 µg/g). Twelve liquids had hexaldehyde concentrations above the LOD (0.036 µg/g) (Table 3).

### 3.7. Tobacco-Specific Nitrosamines

All the samples had nitrosamines concentrations below the LOD (1 µg/g).

### 3.8. Solvents

1,3-butadiene was detected only in H60348 (10 µg/g). The chromatographic resolution was not sufficiently efficient to separate acetaldehyde and ethylene dioxide, but a peak corresponding to these compounds was noticeable in two samples (H60360: 9 µg/g, and H60363: 13 µg/g). Acetone was found in H60348 (20 µg/g) and in H60365 (9 µg/g). The following compounds were found in the following samples only: 1-propanol in H60379 (16 µg/g), 3-hydroxy-2-butanone in H60363 (16 µg/g), 2-methylpropyl acetate (26 µg/g) and methyl, 2-methyl butyrate (12 µg/g) in H60378. 2,3-butanedione was found in three liquids (H60360: 9 µg/g, H60363 : 43 µg/g, and H60378: 12 µg/g). Cyclohexane was detected in two liquids: H60351 (11 µg/g) and in H60367 (6 µg/g). 3-methylbutanal was detected in two products (H60360: 14 µg/g, and H60363: 6 µg/g). 2-methyl-1,3-dioxane and isomers were found in two products (H60378: 41 µg/g, and H60377: 57 µg/g). 1-butanol was detected in two products (H60360:10 µg/g, and H60363: 6 µg/g), whereas ethyl propanoate was detected in three products: H60361 (6 µg/g), in H60378 (123 µg/g), and in H60379 (88 µg/g). 1,1-diethoxyethane was found in H60378 (40 µg/g), H60351 (11 µg/g) and H60380 (23 µg/g). Finally, ethyl acetate was the most important residual solvent present in several samples, in concentrations lower than 100 µg/g except for H60378 (253 µg/g) (Table 4).

**Table 3:** Aldehydes (in µg/g) in 42 bottles of e-liquids, 2013.

Brand	Code	Formaldehyde	Acetaldehyde	Propionaldehyde	Crotonaldehyde	Butyraldehyde	Benzaldehyde	Hexaldehyde
LOD		0.060	0.030	0.043	0.053	0.077	0.035	0.036
Janty	H60339	0.497	0.728	0.043	<0.053	<0.077	<0.035	<0.036
Janty	H60340	0.450	0.545	<0.043	<0.053	0.101	0.036	<0.036
Janty	H60341	0.389	0.591	<0.043	<0.053	<0.077	<0.035	<0.036
Janty	H60342	0.244	0.425	<0.043	<0.053	<0.077	<0.035	<0.036
Janty	H60343	0.884	0.310	<0.043	<0.053	<0.077	0.330	<0.036
Janty	H60344	0.617	0.132	<0.043	<0.053	<0.077	<0.035	<0.036
Janty	H60345	0.138	2.03	<0.043	<0.053	<0.077	<0.035	<0.036
Ecigexpress	H60346	0.161	1.74	<0.043	<0.053	0.186	0.160	<0.036
Ecigexpress	H60347	2.51	0.498	0.066	<0.053	0.178	<0.035	<0.036
Ecigexpress	H60348	0.303	0.539	<0.043	<0.053	<0.077	<0.035	<0.036
Vapor4Life	H60349	0.776	0.507	0.089	<0.053	0.217	40.0	<0.036
Vapor4Life	H60350	0.522	0.737	<0.043	<0.053	<0.077	<0.035	<0.036
Vapor4Life	H60351	0.269	1.49	<0.043	<0.053	<0.077	0.072	<0.036
Totally Wicked	H60352	0.532	0.129	<0.043	<0.053	<0.077	0.821	<0.036
Totally Wicked	H60353	1.13	0.040	<0.043	<0.053	<0.077	<0.035	<0.036
Totally Wicked	H60354	1.25	0.055	<0.043	<0.053	<0.077	<0.035	<0.036
Sedansa	H60355	0.813	1.25	0.167	<0.053	0.164	<0.035	<0.036
Sedansa	H60356	0.409	0.086	<0.043	<0.053	<0.077	0.422	<0.036
Sedansa	H60357	0.590	0.896	0.152	<0.053	0.172	0.247	<0.036
Sedansa	H60358	0.865	0.923	0.074	<0.053	<0.077	0.133	<0.036
Sedansa	H60359	0.681	0.944	0.067	<0.053	<0.077	0.063	<0.036
Johnson Creek	H60360	0.356	2.58	0.122	<0.053	<0.077	0.291	<0.036
Johnson Creek	H60361	2.92	3.08	0.231	<0.053	<0.077	0.245	<0.036
Johnson Creek	H60362	1.97	3.21	0.166	<0.053	<0.077	0.116	<0.036
Johnson Creek	H60363	0.651	2.35	0.261	0.067	<0.077	0.175	<0.036
TECC	H60364	0.467	0.235	<0.043	<0.053	<0.077	0.078	<0.036
TECC	H60365	1.04	0.209	0.189	<0.053	0.478	0.146	0.096
TECC	H60366	0.297	0.299	<0.043	<0.053	<0.077	0.145	0.076
TECC	H60367	0.547	0.559	<0.043	<0.053	1.03	0.582	0.046
TECC	H60368	0.776	0.389	<0.043	<0.053	<0.077	<0.035	<0.036
Intellicig	H60369	0.114	4.05	0.083	<0.053	<0.077	0.581	<0.036
e-cigarettes.fr	H60370	0.257	0.413	<0.043	<0.053	<0.077	0.104	0.068
e-cigarettes.fr	H60371	0.565	0.803	0.049	<0.053	<0.077	0.039	0.105
e-cigarettes.fr	H60372	0.205	0.381	<0.043	<0.053	<0.077	0.060	<0.036
CigLib	H60373	0.274	0.421	<0.043	<0.053	<0.077	0.035	0.089
V2 Cigs	H60374	0.411	0.332	0.045	<0.053	<0.077	0.146	0.115
e-liquide.com	H60375	9.00	3.14	<0.043	<0.053	<0.077	0.145	0.100
Tasty Vapor	H60376	3.52	2.37	<0.043	<0.053	<0.077	305	0.532
Tasty Vapor	H60377	0.441	10.2	0.063	0.084	<0.077	3.70	0.192
Tasty Vapor	H60378	1.88	1.44	<0.043	<0.053	<0.077	9.61	<0.036
e-cig.com	H60379	0.226	0.393	0.047	<0.053	<0.077	0.062	0.132
e-cig.com	H60380	1.95	2.03	<0.043	<0.053	<0.077	0.068	0.081

**Table 4:** Solvents (in µg/g) in bottles of e-liquids, 2013 *.

Code	1,3-Butadiene	Acetaldehyde/Ethylene Oxide	Acetone	1-Propanol	2,3-Butanedione	Ethyl Acetate	Cyclohexane	3-Methyl Butanal	2-Methyl-1,3-Dioxane	1-Butanol	Propanoic Acid, Ethyl Ester	1,1-Diethoxy Ethane	3-Hydroxy-2-Butanone	Acetic Acid, 2-Methylpropyl Ester	Butanoic Acid, 2-Methyl-, Methyl Ester
H60343						72									
H60348	10		20												
H60349						12									
H60351							11					11			
H60360		9			9			14		10					
H60361						8					6				
H60363		13			43	11		6					16		
H60365			9												
H60367							6								
H60369						29									
H60370						76									
H60372						54									
H60376						39									
H60377									41			40			
H60378					12	253			57		123			26	12
H60379				16		11					88				
H60380												23			

* All 42 samples were tested, but samples with no detectable levels of solvents are not shown.

## 4. Discussion

In the absence of therapeutic intention, e‑liquids cannot be considered medications, nor are they considered food products in any country. Rather, they are classified either as tobacco products or as consumer products in countries that have a specific regulation [18]. However, it is important to determine the conformity of these products to the maximum concentrations authorized in relevant categories of products: food, pharmaceuticals, flavors and fragrances.

All the products complied with norms for the absence of micro-organisms. Ethylene glycol and diethylene glycol are not authorized as ingredients in food and pharmaceutical products, but maximum residual limits are allowed, as these substances can be found as contaminants in numerous products. None of the liquids showed a concentration of ethylene glycol and diethylene glycol above these limits (1 mg/g according to FDA and 620 µg/g according to the US Pharmacopeial Convention in 2007) [19,20].

Ethanol (beverage alcohol) is a very common compound found in food and other consumer products. The maximum amount found in the tested liquids was 0.4%, which is authorized if mentioned on the label.

High amounts of hydrocarbons were found in several products from *Tasty Vapor*, in particular alpha-pinene in H60376 (4.8 mg/g) and in H60377 (640 µg/g), at levels higher than the limit of 160 µg/g recommended in finished products. Beta-pinene in H60376 (27 mg/g) was also above the 100 µg/g limit recommended for finished products. Gamma-terpinene in H60376 (11 mg/g) exceeded the 40 µg/g limit recommended for finished products, and benzene 1-methyl-4-(1-methylethyl) (para-cymene) in H60376 (5.5 mg/g) was also higher than the 250 µg/g limit recommended for finished products. These compounds were probably present in the flavors added to these liquids by manufacturing processes, perhaps in an attempt to make the flavoring more intense.

Formaldehyde was detected in all the 42 samples. Formaldehyde concentrations between 0.02 and 10.09 mg/L and acetaldehyde concentrations between 0.10 and 15.63 mg/L have already been reported [21]. Formaldehyde is prohibited in food, and it was probably not added on purpose in the e-liquids, but could be a contaminant present in the ingredients, due to the low quality of raw materials. Of note, formaldehyde also occurs naturally in many food products and in beverages, thus the source might be some natural extracts used as flavorings.

Acrolein and crotonaldehyde should be avoided because they are listed as toxic contaminants in most international legislations (food, environment). For other aldehydes (propionaldehyde, butyraldehyde, benzaldehyde, isovaleraldehyde and hexaldehyde), all of which are approved for use as food flavorings, no sample contained levels higher than those recommended for finished products. Although e-liquids are not considered food products (even if they are consumed as oral mists), compounds such as acetone in samples H60348 and H60365, cyclohexane in samples H60351 and H60367, 1-propanol in H60379, and 1-butanol in H60360 and H60363 were found in quantities higher than their authorized maximum limits as residue in food, as required in 1992 already (5 µg/g for acetone, 1 µg/g for cyclohexane, 5 µg/g for 1-propanol and 1 µg/g for 1-butanol) [15]. Again, these substances may result from the contamination of raw materials, possibly through inadequate purification. The same applies to the two products that contained ethylene oxide (H60360 and H60363). Nitrosamines, 1,3-butadiene and 2-methyl-1,3-dioxane are not cited in most regulations of consumer products or medications, but the carcinogenicity of these compounds is well established [22], and they should not be present in e-liquids at any concentration [23]. We did not detect nitrosamines in any of the 42 e-liquids under scrutiny, but our limit of detection was high (1 µg/g). Our results are in agreement with other studies showing that e-cigarette liquids contain nitrosamines in concentrations lower than the μg/mL range found in tobacco products [24,25]. The origin of 1,3-butadiene and 2-methyl-1,3-dioxane is unclear, but may result from the contamination of ingredients (possibly propylene glycol or glycerine). The amount of acetone is often recommended below 8 µg/g in the finished product and the quantities measured in samples H60365 (9 µg/g) and H60348 (20 µg/g) were above this value.

### 4.1. Acute Oral Toxicity

Although e-liquids are intended to be vaporized and inhaled, the risks associated with ingestion should also be considered. Liquids can be ingested either after deposition of the vapor droplets in the upper aero-digestive tract during normal vaping, or accidentally [26], or intentionally in suicide attempts [27]. Assuming an ingestion of 10 mL of e-liquid, the risk of acute toxicity for components other than nicotine was not significant, because all the estimated concentrations were largely below the known LD50 for various animals (mainly rodents and guinea pigs). Regarding components other than nicotine, the acute oral toxicity of the investigated liquids may not require regulation over and above existing legal requirements or industrial norms. However, it should be mentioned that the proposed scenario of exposure interprets the oral toxicity of detected compounds as ingested compounds that go through the first-pass metabolism, whereas inhaled compounds have direct access to the bloodstream without being metabolized first.

No information concerning the toxicity and maximum thresholds are available concerning cyclohexane 1-methyl-4-(5-methyl-1-methylene-4-hexenyl), 2-methyl-1,3-dioxane, 2-methyl-methylbutyrate, o-tolualdehyde and 2,5-dimethylbenzaldehyde. Therefore, their acute toxicity is not discussed here, but this does not mean that their concentrations found in the liquids are safe.

Thus, the extrapolation of our data to a hypothetical oral ingestion of 10 mL of liquid by an adult (60 kg) should not result in acute toxicity (for compounds other than nicotine), because all the concentrations were at least 480 times below the LD50 for all the compounds under scrutiny. Similarly, the same ingestion by a child (15 kg) should not result in acute toxicity, because all the concentrations were at least 120 times below the LD50 for all the compounds. However, synergistic effects may occur and the acute toxicity of a liquid does not necessarily result from the individual acute toxicity of each compound assessed separately.

### 4.2. Chronic Oral Toxicity Associated with Intended Use

To assess the chronic toxicity associated with intended use, it was assumed that the composition of liquids does not change after being heated and evaporated during e-cigarette use. However, because we did not analyze the vapor composition, our interpretation was only based on the compounds identified in the liquid refills. Moreover, we assumed that the concentration of chemicals in the aerosol was similar to the concentration in the e-liquid. These hypotheses are not necessarily verified. For instance, the levels many toxicant are dependent on battery voltage [28,29]. However, given the relative dearth of published data on the transformation of e‑liquids into aerosols, our assumptions constitute a best-guess scenario that we used for this preliminary evaluation.

For aldehydes, studies have shown that formaldehyde, acetaldehyde, acetone and acrolein are additionally produced during the thermal decomposition of the basic ingredients in e-liquids (propylene glycol and glycerol) [28,30]. Therefore, we did not assess the potential chronic toxicity from oral exposure to these compounds, since this assessment would underestimate the true effects; vapor analysis would be more relevant in the case of aldehydes.

No information concerning the toxicity and maximum thresholds are available concerning cyclohexane 1-methyl-4-(5-methyl-1-methylene-4-hexenyl), 2-methyl-1,3-dioxane, and 2,5-dimethylbenzaldehyde. Therefore, their oral chronic toxicity is not discussed here, but this does not mean that their concentrations found in our sample of e-liquids are safe.

Assuming a chronic exposure of 3 g of e-liquid daily, five terpenic molecules have to be considered: limonene, alpha-pinene and beta-pinene, gamma-terpinene and benzene 1-methyl-4-(1-methylethyl). Terpenic molecules are commonly found in flavors, and they have a relatively low oral toxicity compared to nitrosamines for a same amount. However, the relatively high quantities of these molecules do not guarantee the related products are innocuous.

Limonene was found in five products, but following our chronic toxicity scenario (3 g/day consumption), only H60376 from *Tasty Vapor* (153 mg) was above the MSDI USA limit (13 mg/pers/day), by more than 10 times. [31,32]. The other products contained limonene in quantities below or in the range of MSDI USA. The level of alpha-pinene present in H60376 (14 mg for 3 g ingested) was 6 times above the MSDI-EU limit of 2.2 mg/pers/day cited by EFSA, calculated for a person of 60 kg [32]. Beta-pinene was detected in H60376 at levels corresponding to a daily intake more than 100 times above the MSDI USA limit of 760 µg/pers/day (MSDI EU of 1.3 mg/pers/day) [32], indicating a potential risk from oral chronic exposure. Gamma-terpinene was also found in H60376 at levels leading to a total daily intake more than 100 times above the MSDI USA level of 321 µg/pers/day [32]. The intake of benzene 1-methyl-4-(1-methylethyl) from H60376 represented almost 35 times the MSDI USA limit of 470 µg/pers/day [32]. Consequently, the sample H60376 (*Amaretto Stone Sour* from *Tasty Vapor*) revealed a potential for oral toxicity from chronic daily exposure. For 2-methyl, methylpropanoate, the MSDI-EU limit is 20 μg/pers/day, and this limit would be exceeded from daily exposure to 3 g of sample H60378 by *Tasty Vapor* (36 µg); however, that sample contained levels lower than the maximum recommended in finished product (200 μg/g). 2,3-butanedione (diacetyl) is a diketone associated with the development of respiratory disease; it was present in three samples, with one of them (H60363, *Red Oak Tennessee Cured* by *Johnson Creek*) containing higher than safety levels calculated based on NIOSH-recommended safety limits [33]. For 1,3 butadiene, the non-carcinogen Tolerable Daily Intake (TDI) values for inhalation is 0.57μg/kg/day [34], thus, sample H60348 by *Ecigexpress* would result in marginally safe daily oral exposure (30 μg/day compared to the acceptable level of 34 μg/day in a 60 kg person). No data on safety limits or ADI and TDI values were found for cyclohexane and 2-methyl-1,3-dioxane, while ethyl acetate is of low oral toxicity and all samples were within the recommended maximum values in finished products. For the rest of the hydrocarbons and solvents, the levels of daily exposure were lower than the MSDI values.

For aldehydes, daily exposure to benzaldehyde in all samples was much lower than the MSDI-EU value of 7900 μg/pers/day. The same applies to o-tolualdehyde (MSDI-EU: 1 μg/pers/day; MSDI-USA: 9100 μg/pers/day), m-tolualdehyde (MSDI-EU = 0.85 μg/pers/day) and p-tolualdehyde (MSDI-EU: 160 μg/pers/day; MSDI-USA = 9100 μg/pers/day). Although formaldehyde contents were below the TDI of 150 μg/kg body weight/day defined by WHO for drinking water [35], it was previously mentioned that formaldehyde is a product of thermal degradation and is thus formed during heating and evaporation of the liquid. The same applies for acetaldehyde (which is approved for use in food), and for acrolein and crotonaldehyde [36,37]. No data on MSDI, TDI and EHE exist for propionaldehyde, butyraldehyde, isovaleraldehyde and hexaldehyde. However, these are structural class I chemicals, and for this class, the human exposure threshold for concern is 1800 μg/person/day; none of the samples exceeded this level. For butyraldehyde, the ADI is defined at 0.1 mg/kg/day [38], and none of the samples resulted in exposure to such levels. For hexaldehyde, the estimated limits for intake in USA and EU are 260 and 781 μg/pers/day respectively [39]; again none of the samples would approach the estimated intake limits when consumed at 3 g/day.

It should be emphasized that the exposure will depend on several factors in addition to the liquids themselves, including the e-cigarette model (power, temperature, technical characteristics) and the behavior of the user (duration of use, volume and depth of inhalation, number of puffs). The emission of compounds related to thermal degradation (such as aldehydes) should also be taken into consideration. Therefore, this study represents a preliminary, exploratory approach based on the current knowledge. Clearly, the oral chronic toxicity and the cytotoxicity of e-liquids and e-vapors should be further investigated [40,41].

### 4.3. Strengths and Limitations of This Study

The strengths of our study included the analysis of a large number of some of the most popular brands of e-liquids, and the analysis of two batches of each model for the microbiological tests. One limitation is that some popular brands were not included, which makes our convenience sample of e-liquids not representative of the market in any country. We purchased only commercial liquids, even though home-mixed liquids and random recipes can be of major toxicological concern. Another limitation is that, for cost reasons, we tested only one batch per model for the chemical tests, and therefore could not assess inter-batch variability. Moreover, the data is limited due to a lack of reproduction for outlying data points.

This study was initiated in 2013 based on popular brand data that was collected earlier. With the rapidly changing marketplace, the products analyzed may not represent the brands that currently dominate the market in the USA and Europe.

Although our list of analyzed substances is longer than in most previous reports, analyses of other substances are necessary. These include flavors and fragrances, aroma transporters (propylene glycol, glyceryl mono-, di- and triacetate), food dyes, phthalates and plasticizers (that can migrate from the container during heating and vaporization), metal particles that can detach from imperfect soldering or from the resistance coils [42], allergens and other infectious agents. Moreover, oral toxicity was evaluated based on currently established norms; it is important that direct toxicological assessment is performed, by cytotoxicity experiments on relevant cell cultures, in animals and in clinical studies.

It should be also mentioned that the proposed scenario of exposure interprets the oral toxicity of detected compounds as ingested compounds subjected to metabolism, but inhaled compounds are not metabolized and are potentially more toxic than their metabolites. Therefore, different levels of safety based on route of administration should be considered in the interpretation of such data.

We analyzed refill liquids only, but future studies should analyze the vapors as well, because new substances may be created during the heating and vaporization processes. Tests for delivered dose uniformity and aerodynamic particle size distribution should also be performed, because these tests are mandatory for medications intended to be inhaled.

## 5. Conclusions

None of the products under scrutiny were totally exempt of potentially toxic compounds. As this new market has developed largely outside an appropriate regulatory framework, some manufacturers and vendors apparently lack the adequate know-how about safety.
